# Clinically oriented immune heterogeneity in prostate cancer: emerging targets and strategies

**DOI:** 10.3389/fimmu.2026.1753718

**Published:** 2026-02-12

**Authors:** MingWei Zhan, BinBin Zhao, Junjie Wu, Kai Li, Yibo Chen, Haote Chen, Lin Zhao, Jingyu Zhu

**Affiliations:** 1 Department of Urology, Hangzhou TCM Hospital of Zhejiang Chinese Medical University (Hangzhou Hospital of Traditional Chinese Medicine), Hangzhou, China; 2 Department of Urology, Hangzhou Integrative Medicine Hospital Affiliated to Zhejiang Chinese Medical University (Hangzhou Red Cross Hospital), Hangzhou, China; 3 Department of Urology, Jinling Clinical Medical College, Nanjing University of Chinese Medicine, Nanjing, China; 4 Department of Nephrology, Hangzhou Traditional Chinese Medicine (TCM) Hospital of Zhejiang Chinese Medical University (Hangzhou Hospital of Traditional Chinese Medicine), Hangzhou, China

**Keywords:** bone metastasis and immunotherapy, immune heterogeneity, myeloid-driven immunosuppression, prostate cancer, tertiary lymphoid structures (TLS), tumor immune microenvironment

## Abstract

Prostate cancer (PCa) has long been viewed as an immunologically “cold” malignancy because immune checkpoint inhibitors (ICIs) show limited benefit in unselected patients, particularly after progression to metastatic castration-resistant PCa (mCRPC) or treatment-related neuroendocrine PCa (NEPC). Single-cell and spatial profiling now reveal immune heterogeneity across patients, between lesions, and along the path from localized disease to metastasis. Primary tumors form mosaics of immune-excluded glands, myeloid-suppressed stromal borders, and focal lymphocyte-rich niches with B-cell aggregates and tertiary lymphoid structures (TLS). TLS-high regions represent an actionable “hot minority” resembling inflamed, ICI-responsive cancers, supporting biomarker-guided neoadjuvant or focal immunotherapy. With dissemination, heterogeneity expands across sites; bone metastases become marrow immune organs dominated by suppressive macrophage/monocyte programs and dysfunctional T cells, often driven by the CCL2–CCR6 axis. Standard therapies remodel these ecosystems, creating inflammatory windows yet fostering adaptive resistance. Mechanistically, myeloid-driven, inflammation-coupled rewiring is central to escape: IL-8/CXCR2 signaling and therapy-induced senescence/SASP recruit and polarize suppressive myeloid cells, reinforcing T-cell exclusion and exhaustion. Variable HLA class I loss and hypoxic or metabolic “functional cold zones” add lesion-specific immune invisibility. Clinically, these insights motivate a heterogeneity-aware framework integrating genomic responder subsets with microenvironmental stratification. Barrier-matched strategies include T-cell redirection (PSMA/STEAP1 engagers, bispecifics, CAR-T) and combinations that heat or modulate myeloid cells. Treating immune heterogeneity as a clinical variable enables durable immunotherapy in PCa.

## 1 Introduction

Prostate cancer (PCa) is still one of the most common malignant tumors in men, and its mortality is mainly driven by progression to metastatic castration-resistant prostate cancer (mCRPC) and treatment-related neuroendocrine PCa (NEPC) (1). Although androgen-axis therapy and radioligand therapy have made significant progress, achieving lasting control in late-stage disease remains rare, prompting considerable attention for immunotherapy (2, 3). However, in several clinical trials, immune checkpoint inhibitor (ICI) monotherapy has shown a low objective remission rate in unselected PCa cohorts, further supporting the clinical impression that PCa is usually an immune “cold” tumor (2, 3). This phenotype is closely related to low-baseline T-cell infiltration, weak interferon/antigen-presentation programs in most tumors, and a microenvironment rich in inhibitory myeloid and stromal signals, which limits adaptive immune responses (2, 3).

However, the term “cold PCa” is merely a catchall that masks clinically significant immune diversity. Integrated single-cell and spatial transcriptomic studies revealed substantial differences in immune composition, activation status, and spatial tissue distribution across patients, lesions within the same patient, and disease stages. The common immune rejection area in the primary tumor can be accompanied by microfoci colocated with depleted T cells and an inhibitory myeloid population; in other regions, B cells were found to be aggregated, suggesting the formation of tertiary lymphoid structures (TLS) (4, 5). In addition, the metastatic niche, especially the bone marrow, presents a unique ecosystem with highly enriched myeloid inflammation and suppressed T cells, suggesting that the lesion itself is the central axis of immune heterogeneity and a realistic determinant of treatment response (6). Importantly, immune heterogeneity is dynamic: standard treatment can reshape cytokine and chemokine circuits, change the balance between immune activation and suppression, and thus shape the trajectory of immune escape and drug resistance (3, 4).

**Figure 1:**
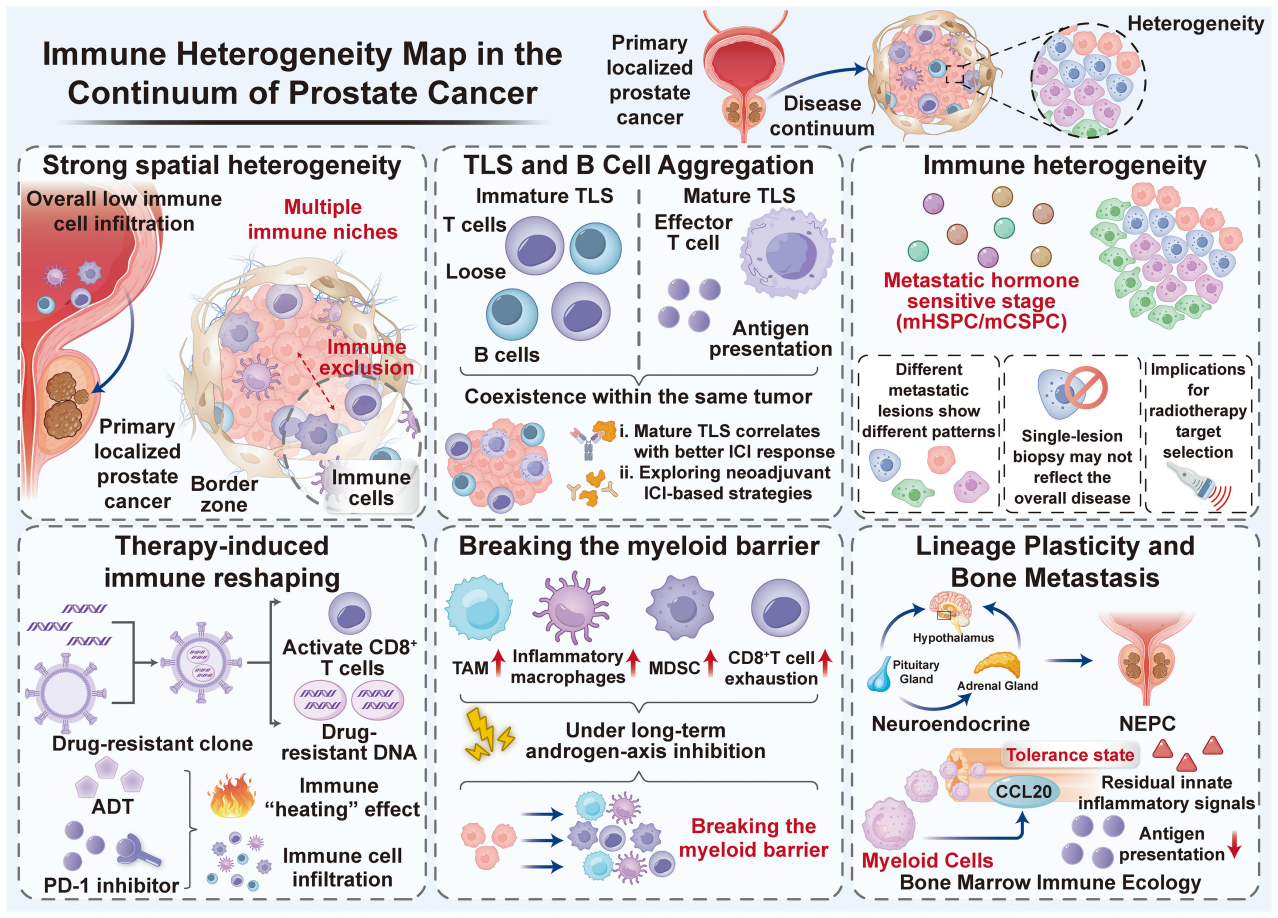
Immune heterogeneity map across the prostate cancer continuum. Conceptual schematic summarizing stage- and site-dependent immune ecosystems, spanning localized disease with pronounced spatial heterogeneity; TLS-associated “hot minority” niches; interlesion divergence in metastatic hormone-sensitive disease; therapy-induced transient inflammatory windows; myeloid-dominant suppressive barriers under chronic treatment pressure; and immune-refractory extremes in bone metastasis and lineage plasticity/NEPC. In bone-dominant niches, excessive CCL20 production by myeloid cells promotes CCR6^+^ T-cell dysfunction, amplifying local immune tolerance and reinforcing systemic immune escape. ADT, androgen deprivation therapy; AR, androgen receptor; TLS, tertiary lymphoid structures; TAM, tumor-associated macrophages; MDSC, myeloid-derived suppressor cells; mCSPC, metastatic castration-sensitive prostate cancer; mCRPC, metastatic castration-resistant prostate cancer; NEPC, neuroendocrine prostate cancer.

The key is that there are still a few “immune-responsive” subgroups of great clinical significance in the overall cold layout. Patients with mismatch repair defect/microsatellite instability (MSI-H/dMMR) or high tumor mutation burden (TMB-high) can obtain durable benefits from pembrolizumab, supporting the use of biomarker-guided ICI in PCa (7). In addition, the CDK12-inactivated mCRPC represents another subgroup defined by the genome, with a higher neoantigen load and enhanced interferon tone, making it more likely to respond to ICI. However, its response remains highly heterogeneous and may depend on the coexisting microenvironment (8). These observations will change the clinical core question from “Is immunotherapy effective in PCa?” Change to “What immune niches exist in patients? How do they evolve under the pressure of treatment? How can we reprogram them into a responsive state?” Based on this, this review systematically integrates the immune heterogeneity data in the continuous PCa spectrum, emphasizes the immune escape mechanism of myeloid–inflammation coupling, and proposes new targets and clinical strategies to transform refractory niches into treatable vulnerabilities. An integrated framework summarizing stage- and site-specific immune ecosystems is presented in **Figure 1**, which serves as a roadmap for the sections below.

## 2 Immune heterogeneity atlas across the prostate cancer continuum

### 2.1 Roadmap: clinical states across PCa progression and representative immune features

To orient cross-disciplinary readers, we briefly define the central clinical states referenced throughout this progression-based review and highlight representative biological and immunological features. Localized hormone-sensitive PCa is organ-confined and typically remains androgen receptor (AR)-driven; across cohorts, the tumor immune microenvironment is often immune-cold on average, with substantial spatial heterogeneity in immune infiltration and functional states (9–11). mCSPC denotes systemic dissemination while disease remains responsive to androgen deprivation therapy (ADT), and metastatic niches—particularly bone—commonly display myeloid-enriched, T-cell-excluded, or dysfunctional immune contexts (8–10). mCRPC is defined by disease progression despite castrate testosterone levels and may manifest as rising prostate-specific antigen (PSA), radiographic progression, and/or new metastases; under the selective pressure of ADT and AR-targeted agents, mCRPC frequently exhibits immunosuppressive remodeling characterized by expanded suppressive myeloid compartments and impaired T-cell effector function (10, 12, 13). Finally, NEPC, including treatment-emergent NEPC, represents lineage plasticity toward an AR-indifferent phenotype and is increasingly recognized in contemporary treatment settings; this phenotype can be associated with altered target expression and additional constraints on effective antitumor immunity, with important implications for immunotherapy responsiveness and trial stratification (14, 15). A concise summary table (**Table 1**) is provided to serve as a roadmap for the stage-specific sections below.

**Table 1:** Clinical states across PCa progression and representative biological/immune features.

Disease state	Clinical definition (one phrase)	Key biology (keywords)	Representative immune features (keywords)
Localized hormone-sensitive PCa	Organ-confined, hormone-sensitive, typically AR-driven	AR dependence; early evolution; spatial heterogeneity	“Immune-cold” on average; marked spatial heterogeneity across glands/stroma; diverse immune neighborhoods in treatment-naïve tissue
mCSPC/mHSPC	Metastatic disease is still responsive to ADT (castration-sensitive)	Systemic dissemination; therapy-naïve to CR	Site-dependent immunity; bone metastatic niche frequently myeloid-enriched; T-cell exclusion/dysfunction common
mCRPC	Progression despite castrate testosterone may manifest via PSA rise and/or radiographic/new metastases.	Selection under ADT/ARSI; resistance programs	Immunosuppressive remodeling; expanded suppressive myeloid compartments; impaired T-cell effector function
NEPC/treatment-emergent NEPC (t-NEPC)	Neuroendocrine transformation often arises under ADT/AR-targeted pressure.	Lineage plasticity; AR-indifferent phenotype; neuroendocrine programs	Often immune-cold; altered antigen presentation/target expression in subsets; implications for immunotherapy responsiveness and stratification

### 2.2 Neuroendocrine transformation and treatment-emergent NEPC: a brief primer

NEPC represents a clinically aggressive phenotype that can arise *de novo* or, more commonly in contemporary practice, as treatment-emergent NEPC (t-NEPC) under selective pressure from androgen deprivation and potent AR-targeted therapies (14). Biologically, NEPC reflects lineage plasticity toward an AR-indifferent state, often accompanied by neuroendocrine differentiation programs and distinct transcriptional/epigenetic regulation; clinically, this transition can coincide with rapid progression, atypical metastatic patterns, and reduced reliance on PSA kinetics (14, 16). Significantly, NEPC evolution may reshape therapeutic vulnerabilities and biomarker performance, including reduced prostate-specific membrane antigen (PSMA) expression in subsets, which can complicate imaging- and target-based strategies (15). From an immunotherapy perspective, NEPC is often considered an “immune-cold” context, and lineage transition may further constrain effective antitumor immunity by altering antigen presentation and tumor–immune interactions, contributing to limited responsiveness to immune checkpoint blockade in many patients (14–16). These features underscore the need for state-aware stratification and rational combinations (e.g., approaches that bypass impaired priming via T-cell redirection, and/or strategies that modulate suppressive myeloid niches) when considering immunotherapy across the PCa continuum (15, 16).

### 2.3 Primary localized PCa: low-average infiltration but high spatial diversity

Limited PCa is often considered to have few immune cells, but the integrated single-cell and spatial transcriptomic maps show significant intratumoral variation underlying the “low overall invasion”. In the same prostate sample, the immune rejection gland region coexisted with a stromal interface rich in T cells and myeloid cells. The ligand–receptor network suggests that organized epithelial–fibroblast–myeloid cell crosstalk maintains local immune stagnation (17–19). These spatial neighborhoods are clinically significant because sampling can miss a small range of immunoreactive foci, and the dominant barriers in different regions may differ (physical rejection *vs*. functional inhibition). Therefore, even in early PCa, it should be regarded as a puzzle of multiple immune niches rather than a heterogeneous cold tumor.

### 2.4 TLS and B-cell aggregates define an actionable “hot minority” in primary disease

A considerable number of primary tumors contain B-cell clusters and TLS. Recent spatial studies have emphasized that TLS is also heterogeneous in location and maturity. Immature TLS showed loose B/T-cell aggregation, while mature TLS showed a structure similar to the germinal center, accompanied by stronger antigen presentation and effector T-cell characteristics; they can coexist in the same tumor, suggesting different local developmental trajectories (20–22). Clinically, the TLS-high/mature TLS region is similar to the immune-inflammatory microenvironment in ICI-responsive tumors, supporting the use of neoadjuvant or local immunotherapy in carefully selected patients for a limited period, rather than for all patients.

### 2.5 Metastatic hormone-sensitive PCa: dissemination amplifies interlesion heterogeneity

When PCa enters metastasis but is still sensitive to hormone, the immune diversity expands along a new axis—the differences between different lesions are significant. Different metastatic sites within the same patient often exhibit distinct immune density and composition, suggesting that diffusion is accompanied by site-specific microenvironment imprinting and a “Founding” immune state (17, 18). This has practical consequences: a single biopsy lesion cannot represent the overall immune landscape of patients, and treatment response may also be inconsistent across lesions. Therefore, multisite heterogeneity should be considered in the early joint scheme, especially when selecting monitoring or irradiation lesions.

### 2.6 Therapy-induced immune remodeling creates transient windows of vulnerability

Standard treatment will actively reshape the immune state, rather than only select drug-resistant clones. ADT can rapidly transform some lesions into a more inflammatory environment, increase the number of activated CD8^+^ T cells, and expand inhibitory regulatory T (Tregs) and myeloid cells (23, 24). In the metastatic castration-sensitive stage, the combination of programmed cell death protein 1 (PD-1) blockade and ADT can induce intense immune infiltration and interferon-related transcription programs in some patients, demonstrating the available “heating effect” (23). The treatment sequence is also important: a sequential study of ADT and vaccine shows that different sequences can alter the balance between the initiation of effects and compensatory inhibition (25). This evidence indicates that the immune heterogeneity of PCa is dynamic and treatment-dependent, and that the inflammatory window in the early posttreatment period is a key opportunity for rational combination therapy.

### 2.7 mCRPC: chronic treatment pressure drives myeloid-inflamed, T-cell-exhausted ecosystems

After long-term androgen axis inhibition, the immune landscape usually evolves toward more potent inhibition and greater heterogeneity. Multigroup studies showed an inflammatory monocyte/MDSC-like status and an amplification of the inhibitory tumor-associated macrophage(s) (TAM) program, while CD8^+^ T cells were depleted and spatially excluded from the gland (18). Importantly, mCRPC is not just “colder” but is differentiated into multiple myeloid-dominated inhibition niches, each with distinct reversibility and specific inhibition circuits. In clinical transformation, this means that T-cell-oriented therapies (ICI, vaccines, T-cell connector (TCE), etc.) often need supporting strategies to dismantle the lesion-specific myeloid barrier.

### 2.8 Lineage plasticity and bone metastasis represent immune-refractory extremes with site-specific rules

CRPC generates treatment-related NEPC through lineage plasticity, which is usually characterized by sparse adaptive immune infiltration and reduced antigen presentation. However, residual congenital inflammatory changes can still be observed across different lesions (26). Bone metastasis is the leading lethal site, forming a more highly specialized immune organ. Single-cell analysis of bone metastases revealed an inhibitory myeloid ecology of bone marrow imprinting, and depleted T cells were embedded within a chemokine field that promoted dysfunction (8). The recurrent and operable pathway is the CCL20–CCR6 axis: the excessive production of CCL20 by myeloid cells in bone marrow drives CCR6^+^ T cells into a tolerant state; blocking this axis can restore T-cell activity and improve survival in the model (8, 27, 28). In addition, heterogeneity in HLA class I expression across lesions can regulate immune visibility, which may explain why, even in a population selected for biomarkers, some metastatic lesions remain treatment-resistant (29). Clinically, these findings support bone-specific immune stratification and suggest that effective systemic immunotherapy must be tailored to the biology of metastatic sites. Together, these stage- and site-dependent immune ecosystems can be viewed as an integrated continuum; an overview is provided in **Figure 1**.

### 2.9 Histological variants: divergent immune landscapes of acinar and ductal architectures

PCa significantly dictates its tumor immune microenvironment. Conventional acinar adenocarcinoma (AA) typically represents an “immunologically cold” phenotype, characterized by limited T-cell infiltration and a “lymphocyte-excluded” pattern, where immune cells are physically sequestered in the periglandular stroma (30). In contrast, ductal adenocarcinoma (DA) and intraductal carcinoma of the prostate (IDC-P) exhibit a more aggressive and immunosuppressive niche. Recent spatial profiling has revealed that DA, despite higher immune cell densities in some instances, often harbors a higher proportion of dysfunctional CD8^+^ T cells and M2-type macrophages, driven by genomic instabilities such as PTEN loss and BRCA2 alterations (31). Furthermore, IDC-P has been identified as a distinct “hot” yet highly suppressed niche, characterized by significantly increased recruitment of Tregs and myeloid-derived suppressor cells (MDSCs) compared to adjacent acinar components (32, 33). This lineage-specific heterogeneity suggests that, while AA may require strategies to “heat up” the tumor, aggressive variants such as DA and IDC-P may necessitate combination therapies targeting myeloid-driven suppression or T-cell exhaustion (34).

## 3 Myeloid-driven and inflammation-coupled mechanisms of immune evasion and resistance

### 3.1 Myeloid heterogeneity forms the suppressive backbone of PCa TME

Myeloid cells are the most abundant and transcriptionally diverse immune components in PCa, with limited metastatic potential and therapeutic tolerance. Single-cell and spatial analysis distinguished a variety of TAM states (including SPP1^Hi, inflammatory, interferon-responsive, etc.), MDSC-like monocytes, and neutrophil/tan programs. These subgroups occupied distinct spatial neighborhoods and exerted distinct immune-regulatory effects (18, 35, 36). Functionally, they synergistically inhibit antigen presentation, suppress T cells, and produce inhibitory metabolites in a lesion-specific manner, which explains why “myeloid-high PCa” itself is also a heterogeneous clinical entity. Space studies further showed that the myeloid-rich areas were often aligned with fibroblasts and ECM-rich, high-density matrix areas, forming a physical and biochemical barrier that captured T cells at the edge of the tumor rather than allowing them to enter the gland (17–19, 36). The diversity of multiple myeloid systems determines the reversibility of different lesions to targeting strategies. It also explains why some lesions in the same patient respond to immunotherapy, while others remain tolerant.

### 3.2 Inflammatory chemokine circuits—especially IL-8/CXCR2—drive myeloid recruitment and T-cell dysfunction

One of the key findings in recent years is that androgen axis destruction and tumor stress can amplify the inflammatory cytokines/chemokines program and actively recruit inhibitory myeloid cells. Interleukin (IL)-8 (CXCL8) and its receptors, CXCR1/2, serve as core mediators linking inflammation and immune escape: IL-8 promotes neutrophil/MDSC chemotaxis, supports tumor cell survival and invasion, and is associated with androgen-independent progression (37–39). In the transformation queue, castration or AR blockade can enhance IL-8 signaling, leading to increased myeloid infiltration, decreased antigen-presenting tone, and increased immunosuppression (38, 40). This establishes a clinically operable logic. Although some lesions are “more inflamed” after treatment, the inflammation tends to be myeloid rather than T-cell-mediated, thus turning the potential “heat” into adaptive resistance. Moreover, IL-8/C-X-C chemokine receptor 2 (CXCR2) upregulation was not consistent across all lesions, further indicating that inflammatory reprogramming was an essential source of heterogeneity between metastases.

### 3.3 Therapy-induced senescence and SASP create a feed-forward myeloid inflammatory loop

In addition to classical cytokines, treatment-induced cellular senescence is increasingly regarded as the driving factor of mCRPC immune heterogeneity and drug resistance. Aging tumor or stromal cells release an aging-related secretory phenotype (SASP) rich in proinflammatory factors, which recruits and polarizes myeloid cells into an immunosuppressive state (41–43). A landmark clinical transformation study showed that age-related myeloid inflammation promoted the progression of metastatic CRPC and blocked resistance to AR inhibition of myeloid chemotaxis, which can reduce inhibitory infiltration and bring lasting benefits in some patients (37). Conceptually, this supports the “inflammation switch” model of PCa resistance: treatment triggers senescence/SASP → SASP recruits inhibitory myeloid → myeloid, in turn, enhances tumor survival and immune escape. It is worth noting that aging is reversible in some situations, meaning that blocking the SASP–myeloid circuit may reopen the immune control window and restore the sensitivity of subsequent immune activation (41, 43).

### 3.4 Bone metastasis exemplifies myeloid-T–T-cell crosstalk as site-specific immune tolerance

The bone marrow niche will amplify the process described above. In bone metastases, macrophages and inflammatory monocytes are dominant, and depleted T cells are embedded within a chemokine field that renders them dysfunctional (8, 26, 27). The recurrent pathway is CCL20–CCR6 signaling: intramedullary myeloid cells produce CCL20, while CCR6^+^ T cells are tolerant; genetic or pharmacological blockade can restore T-cell activity and improve the survival of the model (8, 26). The clinical inference is that bone marrow-dominant lesions naturally tend to fail immunotherapy unless bone-specific myeloid circuits are neutralized at the same time. This also provides a mechanism explanation for the inconsistent response between lesions in systematic treatment, highlighting the necessity of using the metastasis site as a stratified variable in the experiment.

### 3.5 Antigen-presentation loss and metabolic suppression layer further heterogeneity onto myeloid dominance

Even in the presence of T cells, many PCa lesions still showed decreased HLA class I expression, limiting antigen presentation and facilitating immune escape. This phenomenon differs significantly across lesions and may be reversible via the epigenetic pathway (29, 44–46). Simultaneously, hypoxia and changes in lipid/adenosine metabolism form a spatially limited “functional cold zone”, which inhibits T-cell killing and cooperates with myeloid suppression (36). These immune stealth and metabolic binding layers explain why it is not enough to improve immune infiltration alone, unless the dominant inhibitory circuit (usually the medullary center, maintained by inflammation) is disassembled synchronously. Clinically, this strengthens the “two-step” strategy: immune visibility/effect fitness is first restored, and immune effect immunity is then amplified, rather than assuming that deep heterogeneity can be overcome simply by releasing checkpoints.

The circuits highlighted in this section are readily translatable to combination-trial design and correlative biomarker strategies. For instance, IL-8/CXCR2-driven neutrophil and MDSC recruitment can be monitored in peripheral blood (e.g., circulating IL-8 levels and the neutrophil-to-lymphocyte ratio), and elevated systemic or tumor-associated IL-8 has been linked to reduced clinical benefit from PD-(L)1 blockade, supporting its use as a clinically relevant biomarker/readout (40). These blood- and tissue-based readouts also support pharmacodynamic endpoints for CXCR1/2 blockade or IL-8-axis targeting, including rational combinations with ICIs (47, 48). In parallel, senescence/SASP-associated inflammatory programs can reinforce suppressive myeloid states and impair antitumor T-cell function, providing additional rationale for senescence-targeting (senomorphic/senolytic) components in combination regimens (49).

Likewise, lesion-level heterogeneity in antigen presentation—particularly human leukocyte antigen class I (HLA-I)/B2M loss—has been implicated in acquired resistance to PD-1 blockade, underscoring the need to assess baseline antigen presentation capacity when selecting immunotherapy strategies (50). Such profiling can help guide the choice between priming-dependent approaches (e.g., ICI or cancer vaccines) versus priming-bypassing strategies (e.g., bispecific T-cell engagers or chimeric antigen receptor T cell(s) (CAR-T) therapies) (51). Finally, hypoxia- and adenosine-associated immunometabolic suppression and TGF-β-driven immune exclusion provide a translational rationale for incorporating adenosine/CD73-axis or TGF-β-targeting add-ons into rational combinations to overcome “functional cold” barriers (52, 53).

## 4 Clinically actionable stratification and emerging targets/strategies

To translate immune heterogeneity into clinical decision-making, it is first necessary to recognize that, although the proportion of PCa patients who respond is small, distinct subgroups can benefit from ICI. At present, clinical practice supports the use of pembrolizumab in mCRPC defined by biomarkers, particularly in patients with MSI-H/dMMR and TMB-high. This is based on the tumor-agnostic approval and the durable PSA and imaging responses observed in the PCa-specific cohort (7, 54). CDK12-inactivated tumors represent another genomically enriched subgroup, characterized by a higher neoantigen load and a stronger interferon tone. Nevertheless, responses remain highly heterogeneous and may depend on the coexisting microenvironment (8). Overall, these data suggest that the apparent failure of ICI in “all patients” more likely reflects population heterogeneity rather than an absolute lack of immunotherapy efficacy.

In addition to genomic stratification, microenvironment-based typing is emerging as the next level of clinical operability. Spatial and single-cell studies in the second and third sections showed that some lesions were driven by TLS-high/CXCL13 programs, resembling an immune-inflammatory ecology, whereas others are characterized by myeloid dominance, immune exclusion, or metabolic inhibition (20–22, 36). Clinically, these observations support a “two-track framework”: (I) tumors with TLS enrichment or pre-existing lymphocyte infiltration may be prioritized for ICI-containing regimens or vaccine/ICI combinations; and (II) myeloid-inflammatory lesions may require relief of suppressive barriers (e.g., targeting chemotaxis, CSF1R, CXCR2, adenosine, or TGF-β pathways) before T-cell therapy can be effective (37–39). Another practical corollary is that the metastatic site should be incorporated as a stratification variable. Bone-dominant disease is often accompanied by CCL20–CCR6-associated T-cell dysfunction and myeloid tolerance. If the bone microenvironment is not cotargeted, responses in extraskeletal lesions are also diminished (55, 56). Taken together, these genomic- and niche-informed strata can be operationalized into a unified decision framework, as summarized in **Table 2**.

**Table 2:** Clinical decision framework based on immune heterogeneity in prostate cancer.

Stratification axis/immune ecosystem	Key features (what you would diagnose)	Dominant barrier	Matched clinical strategy	Representative agents/examples
Genomic ICI-responsive mCRPC subgroups	MSI-H/dMMR; TMB-high	High neoantigen load → preexisting inflamed potential	ICI monotherapy or ICI-anchored combos in biomarker-selected patients	Pembrolizumab for MSI-H/dMMR or TMB-high mCRPC
Genomic “enriched but heterogeneous” subgroup	CDK12-inactivated tumors; higher neoantigen burden and IFN tone	Mixed immune visibility + coexisting suppressive TME	ICI in CDK12-selected patients, often needing TME-targeting add-ons	PD-1/PD-L1 ± CTLA-4 blockade in CDK12-loss mCRPC
TLS-high/CXCL13-driven lesions (“hot minority”)	B-cell aggregates and tertiary lymphoid structures; lymphocyte-primed niches	Relatively inflamed niches; not primarily exclusion	Prioritize ICI-containing regimens or vaccine + ICI	PD-1/PD-L1 blockade; cancer vaccines + ICI
Myeloid-dominant, immune-excluded, metabolically suppressive lesions	High TAM/MDSC/neutrophil programs; T-cell trapped at margins; hypoxia/metabolic cold zones	Myeloid suppression + T-cell exclusion/exhaustion	Desuppress/”heat” first, then T-cell therapies	Chemokine blockade (IL-8/CXCR2), CSF1R inhibitors, adenosine pathway inhibitors, TGF-β blockade
Bone-dominant metastatic niche (marrow-imprinted)	Suppressive macrophage/monocyte ecology; dysfunctional CCR6^+^ T cells; CCL20–CCR6 axis	Site-specific myeloid tolerance	Treat metastatic site as a stratifier; cotarget bone niche circuits	CCL20–CCR6 pathway blockade (preclinical/early translational); bone-tailored myeloid-modulating combos
Antigen-presentation-low/NEPC/HLA-I loss lesions	Low HLA-I/weak priming; lineage plasticity (NEPC)	Insufficient priming/antigen visibility	T-cell redirection to bypass priming/HLA defects	PSMA TCE/BiTE (acapatamab/AMG-160); STEAP1-TCE (xaluritamig/AMG-509); CAR-T
Cold/excluded lesions needing “heating”.	Low-baseline T-cell infiltration; therapy creates transient inflammatory windows	Cold state with risk of myeloid rebound	Rational heating combinations timed to exploit windows	PARP inhibitor + ICI; RT or PSMA-RLT + ICI; ADT/ARSI-sequenced ICI
Nonclassical checkpoints/broad antigens	B7-H3 (CD276) highly expressed across stages; linked to immune evasion	Alternative inhibitory signaling and antigen heterogeneity	Target B7-H3 axis with multimodal immunotherapies	B7-H3 ADCs; bispecific costimulators; B7-H3 CAR-T

Emerging treatment strategies can be organized according to the heterogeneous barriers they aim to overcome. T-cell redirection therapies—TCEs, bispecific antibodies, and CAR-T—aim to bypass endogenous hypopriming or loss of HLA-I and to physically recruit effector T cells to tumor antigens. PSMA-targeted BiTE, such as acapatamab (AMG-160), has shown encouraging early activity, with CRS remaining manageable in heavily pretreated mCRPC, thereby supporting the feasibility of this strategy (57, 58). STEAP1-targeted TCEs, such as xaluritamig (AMG-509), provide a second important antigen axis and have resulted in deep PSA declines in phase I studies, further demonstrating that antigen-specific redirection can remain effective even in immunologically cold tumors (57, 59). These approaches may be particularly suitable for NEPC or lesions with low antigen presentation, where immune checkpoint blockade alone is often insufficient (26, 29).

Another strategy aims to “heat” cold or immune-rejected lesions to unlock the effects of ICI or T-cell redirection. Rational combination strategies include PARP inhibitors + ICI (leveraging DNA damage → sting → type I IFN signal), radiotherapy or PSMA radioligand therapy + ICI (inducing immunogenic cell death and potential bystander effects), and carefully designed ADT/ARSI sequencing with ICI to exploit the transient inflammatory window before myeloid rebound becomes dominant (2, 3, 23–25). A central clinical insight emerging from immune heterogeneity research is that the same combination strategy will not be equally effective across all lesions. Regimens incorporating myeloid-targeting or regulatory components are more suitable for patients with myeloid-rich or bone-dominant lesions, whereas patients with high TLS content may benefit from a simpler ICI-anchored approach.

Nonclassical checkpoint antigens provide additional clinical leverage. B7-H3 (CD276) is highly expressed across multiple stages of PCa and is associated with immune escape and poor prognosis. It is currently being exploited in ADCs, bispecific costimulatory molecules, and CAR-T platforms to address antigen heterogeneity (60–62). In general, these emerging targets and strategies support a clinically oriented view that PCa comprises multiple coexisting immune niches. Only by accurately matching treatment to the dominant obstacles within each niche, rather than treating PCa as a single immune state, can durable therapeutic benefit be achieved.

Finally, despite the clinical potential of the aforementioned strategies, current stratification tools face significant hurdles. Liquid biopsies, while offering noninvasive monitoring, may fail to capture the full spatial complexity of multifocal primary tumors or heterogeneous metastatic niches (63). Similarly, functional imaging modalities such as PSMA-PET/CT can underestimate aggressive, PSMA-low, or neuroendocrine-like clones, resulting in incomplete immune mapping (64, 65). To address these challenges, future efforts must move toward multimodal stratification frameworks. By integrating AI-powered digital pathology, longitudinal ctDNA/CTC signatures, and radiomics, it is possible to construct a more dynamic and holistic “immune–clinical” map (66, 67). This integrated approach will be essential for identifying patients who require complex combinations, such as myeloid-modulating agents paired with T-cell redirectors, thereby moving beyond the “one-size-fits-all” immunotherapy paradigm (68, 69).

Beyond multimodal clinical stratification, emerging experimental and computational multiomics platforms will further enable mechanistic dissection of how TME composition and spatial organization shape tumor phenotypes and gene-regulatory responses to microenvironmental cues. First, multimodal AI frameworks can translate routinely available histology into higher-dimensional, population-scale representations of the tumor immune microenvironment, enabling *in silico* modeling of spatial protein organization and virtual cohort analyses of TME–phenotype associations (70). Second, large-scale genetic perturbation coupled with multimodal single-cell readouts provides a systematic approach to infer causal gene-regulatory networks and cell-state transitions underlying plasticity and therapy resistance (71). Third, single-cell approaches that directly link targeted genotypes to chromatin accessibility states enable mapping of clonal genetic programs to regulatory landscapes, offering a mechanistic bridge from tumor genotype to heterogeneous therapeutic responses (72). Integrating these modalities with spatial transcriptomics/proteomics and longitudinal sampling should accelerate identification of actionable TME–tumor regulatory circuits and improve rational design of barrier-matched combination strategies.

## 5 Conclusion

Immune heterogeneity provides a clinically valuable framework to explain why prostate cancer is typically unresponsive to immune checkpoint blockade in unselected populations, yet can show meaningful benefit in defined contexts. Across the disease continuum, prostate cancer is better viewed as a dynamic mosaic of immune niches that vary by stage, lesion, and metastatic site, in which the dominant barrier may include physical exclusion, myeloid-driven suppression, impaired antigen presentation, or marrow-imprinted tolerance in bone. Mechanistically, therapy- and stress-associated inflammatory rewiring—often converging on suppressive myeloid programs and T-cell dysfunction—supports a barrier-matched, two-step therapeutic logic: first, identifying and dismantling the dominant suppressive circuit within each niche, and then amplifying antitumor immunity using appropriately selected immunotherapies, including checkpoint blockade and T-cell-redirecting strategies that can bypass priming constraints. Moving forward, actionable stratification should integrate genomic responder subsets with microenvironmental and site-specific states, supported by multilesion assessment and longitudinal, multimodal monitoring. Treating immune heterogeneity as a clinical variable will enable more rational trial design and improve the likelihood of durable immunotherapy benefit in prostate cancer.
